# Understanding how dogs encourage and motivate walking: cross-sectional findings from RESIDE

**DOI:** 10.1186/s12889-016-3660-2

**Published:** 2016-09-29

**Authors:** C. Westgarth, M. Knuiman, H. E. Christian

**Affiliations:** 1 Department of Epidemiology and Population Health, Institute of Infection and Global Health, and School of Veterinary Science, University of Liverpool, Leahurst, Chester High Road, Neston, Cheshire, CH64 7TE UK; 2 School of Population Health, The University of Western Australia, 35 Stirling Highway, Crawley, Perth, WA 6009 Australia; 3 Centre for the Built Environment and Health, School of Sport Science, Exercise & Health, The Univers’ity of Western Australia, 35 Stirling Highway, Crawley, Perth, WA 6009 Australia; 4 Telethon Kids Institute, The University of Western Australia, 100 Roberts Road, Subiaco, Perth, WA 6008 Australia

**Keywords:** Motor activity, Dogs, Walking, Health behavior, Motivation, Physical activity, Psychology

## Abstract

**Background:**

Many people live with dogs but not all walk with them regularly. This study examines the demographic and behavioural factors that contribute towards owners reporting having a strong sense of encouragement and motivation to walk provided by their dogs, which we call ‘the Lassie effect’.

**Methods:**

Data was collected from 629 dog owners participating in the RESIDE cross-sectional survey in Perth, Western Australia. Multivariable logistic regression analyses of factors associated with two separate outcome survey items ‘Dog encouragement to walk’ (how often dog encouraged me to go walking in last month) and ‘Dog motivation to walk’ (Having a dog makes me walk more).

**Results:**

Owning a larger dog; having an increased level of attachment to dog; knowing dog enjoys going for a walk; believing walking keeps dog healthy; and having high social support from family to go walking, were positively associated with both outcomes ‘dog encouragement to walk’ and ‘dog motivation to walk’. Conversely, reporting the presence of children at home; that the child is the main person who walks with the dog; and perceiving dog-specific barriers to walking with dog daily; were negatively associated with both outcomes. In addition, ‘Dog motivation to walk’ only was positively associated with a belief walking reduces barking, and negatively with owning a dog that is overweight or a dog that is too old/sick. Reporting that the spouse/partner is main person who walks with the dog was also negatively associated with ‘dog motivation to walk’, as was increased perceived access to public open spaces with dog-supportive features.

**Conclusions:**

There are both dog and owner factors that are associated with an owner’s sense of encouragement, and motivation to walk the dog, which in turn has been found to be associated with dog waking behaviour. These factors may be targeted in future interventions to increase and maintain physical activity levels of both people and pets.

## Background

Dog ownership is associated with increased physical activity through walking [1]. The benefits arising from owning and walking a dog are of particular public health interest due to their potential for a positive and long-term sustainable effect on the maintenance of physical activity behaviour [2–4] and associated reduction in cardiovascular risk [5]. Considering the significant proportion of people who own or have access to dogs (for example up to 47 % of households own dogs in the US [6], 25 % in the UK [7], and 39 % in Australia [8]), dog walking is a sustainable preventive medicine strategy with wide reach. Further, a recent review of the association between dog ownership and physical activity identified that approximately 40 % of people who live with a dog are not walking with them, and more could be walking with their dog more regularly [1]. In view of the considerable investment in physical activity interventions, investigation of the mechanisms through which dogs facilitate increased physical activity is required to identify feasible dog walking intervention strategies.

A review of the correlates of dog walking found that the key factor associated with people walking with their dog centres around the dog-human relationship [9], however studies have used inconsistent methodology to measure this. For example, Japanese dog walkers had higher levels of ‘attachment’ to their dogs than owners who did not walk their dog [10], as did UK children [11], and in a sample of Australian owners and dogs, higher scores on a dog-owner interaction scale was associated with more frequent exercise [12]. A construct called ‘dog obligation’ has also been used as a measure to describe an owner’s sense of obligation and/or responsibility to walk their dog regularly and a feeling that their dog pressures them to take it for a walk [13], and has been shown to mediate the relationship between dog ownership and physical activity [13, 14]. However the term ‘obligation’ suggests that dog walking is only done out of sense of duty, rather than dog walking being a more positive motivating experience. Supporting this, the authors who originally introduced the idea of ‘dog obligation’ have more recently suggested that it is better termed ‘responsibility’ [15]. Whilst the idea of feeling ‘obliged’ or ‘responsible’ to walk your dog may indeed include feelings of guilt [16], it might also encompass constructs such as valuing exercise for the dog [17]. Other studies found that dog owners who perceive that their dog provides motivation and/or social support for walking [16, 18, 19], or similarly, feel that the dog provides encouragement to walk [14], are more likely to walk regularly with the dog. This motivation provided by the dog to walk may also prove to be an important intervention strategy because targeting the canine need for exercise, rather than the human need, may have greater success in increasing owner activity [20].

Overall, there appears to be something special relating to the motivation, encouragement, obligation or social support (however described in previous studies) for walking that a pet dog can provide compared to other types of motivators or social support mechanisms for physical activity. These concepts describe an aspect of the dog-human relationship that we refer to as ‘the Lassie effect’ due to the iconic television character’s ability to perform life-saving acts.

Whilst there is mounting evidence of the positive influence of the Lassie effect on dog walking behaviour it is not known what factors facilitate or are barriers to the Lassie effect in supporting human physical activity [1, 9]. One issue with the research so far is the lack of association observed between dog walking and dog demographic and behavioural factors (e.g., size, presence of behavioural problems, and number of dogs owned) that we might expect to affect walking. Previously it has often been found that once the Lassie effect measures are included in any modelling, dog demographic and behavioural factors are no longer associated with dog walking [9], not even ‘knowing that the dog enjoys going for a walk’ [18, 19].

**Fig. 1:**
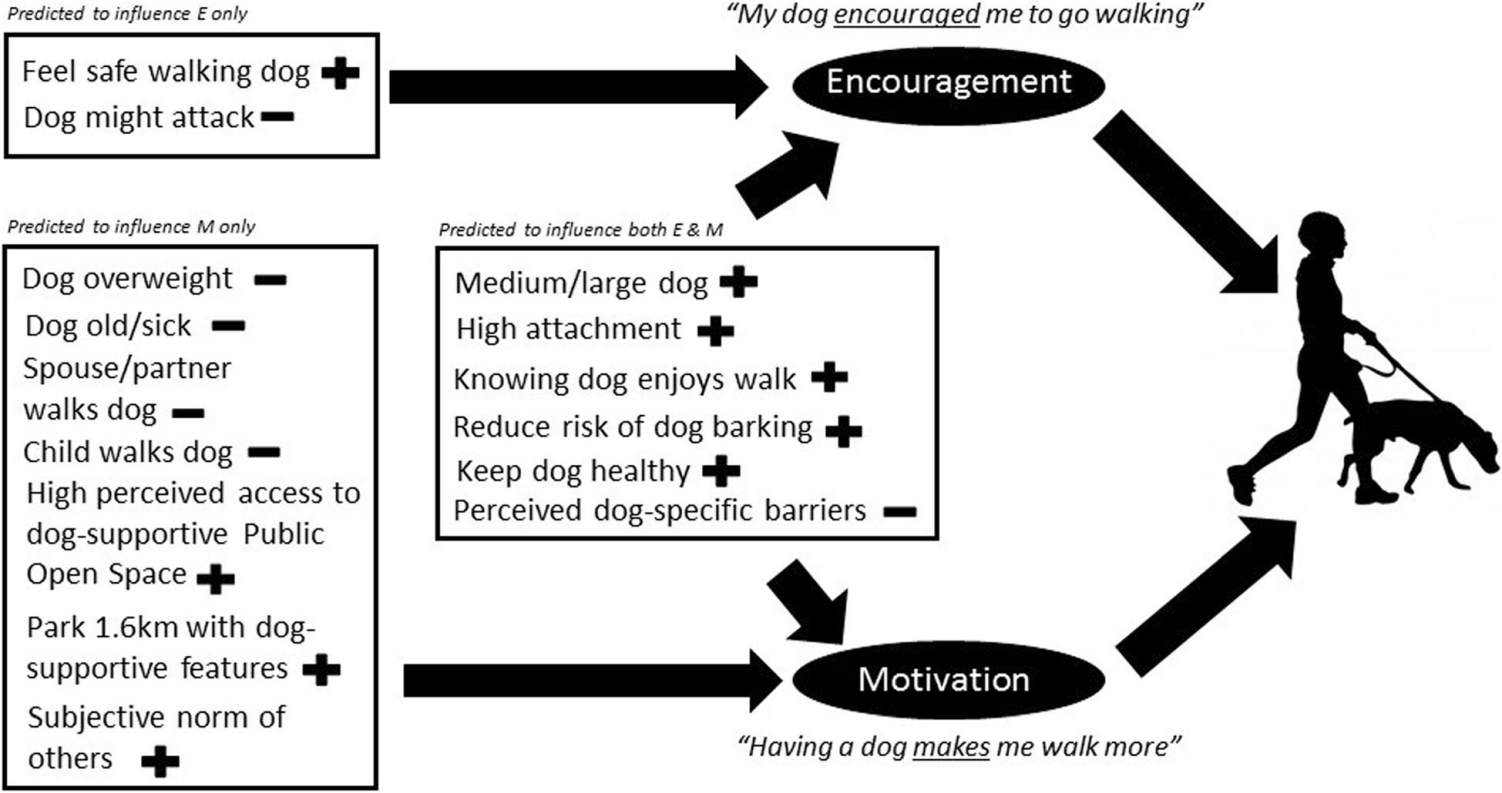
Conceptual map of hypothesised factors associated with encouragement and motivation provided by dogs for walking in the RESIDE dataset

We hypothesised that these dog demographic and behavioural factors are important, but act upon dog walking behaviour through their ability to create the Lassie effect, which then positively affects walking behaviour. A conceptual model relating to our proposed dataset is presented in Fig. 1. Understanding the factors that contribute to the Lassie effect will help inform the design of interventions that use the pet dog to initiate, increase and maintain walking behaviour. Thus the aim of this study was to re-examine a well-characterised dataset regarding dog ownership and physical activity, and model dog-related factors that contribute towards this strong sense of encouragement and motivation to walk the dog.

## Methods

The study dataset has been previously reported in detail [4, 18, 19, 21, 22]. In brief, the RESIDential Environment (RESIDE) project is a longitudinal natural experiment of 1813 people building homes in 73 new housing developments across metropolitan Perth, Western Australia (recruited Sep 2003-Mar 2005). Further information on RESIDE methods are available elsewhere [23, 24]. The validated Dogs And Physical Activity (DAPA) Tool [22] was included as part of the second RESIDE survey. A total of 629 dog owners were included in our new analyses. Ethical approval was provided by the University of Western Australia, and all participants provided written consent. Ethical approval for this new analysis was obtained in 2013 and analysis performed in 2014.

### Lassie effect outcome variables

Although these may appear similar concepts, 1) ‘Dog provides motivation to walk more’; and 2) ‘Social support provided by dog to walk’, have both been shown in previous studies to be independent predictors of dog walking outcomes, indicating inherent differences [18, 19]. Therefore both were chosen to be modelled in this study, this time as outcomes.

The construct ‘social support provided by dog to walk’ that has previously found to be associated with dog walking [18, 19, 22] includes two items relating to how often the dog ‘went walking with me’ and ‘gave me encouragement to go walking’ in the past month. This format was developed in line with other standard social support variables. For this new analysis we chose to use only the item ‘dog gave me encouragement to go walking’ (5 point Likert scale never to very often-3 or more times per week), because this separated the part of the construct that refers specifically to the feelings of encouragement. The new outcome has been named ‘Dog encouragement to walk’ and recoded as a 2 level variable; ‘Very often’ (3 or more times per week) versus ‘Less than very often’ (less than 3 times per week).

The previous predictor variable ‘Dog provides motivation to walk more’ [18, 19, 22] was created using findings from focus groups [16]. It was measured on a 5-point Likert scale (strongly disagree to strongly agree) by the item ‘Having my dog(s) makes me walk more’. The use of the word ‘make’ in the statement suggests that this factor has similarities to the sense of obligation or responsibility as described by other studies [13–15]. For analysis it is coded as a 2 level variable; ‘disagree/neutral’ versus ‘agree’ and named ‘Dog motivation to walk’.

### Independent variables

The independent variables consisted of dog and owner demographics and owner cognitive factors and beliefs which have been described previously [18, 19] (see Tables 1 and 2). In brief, these included dog-related factors such as size and level of attachment to the dog, and whether the dog was overweight or old/sick. They also included Theory of Planned Behaviour constructs of attitude, subjective norm, and perceived behavioural control relating to dog-specific items. The variable ‘Dog-specific barriers to walking daily’ consisted of items pertaining to concerns regarding difficulty of walking two dogs, their dog being unfriendly or difficult to control, and fear of other people’s dogs. Perceived access to public open spaces with dog-supportive features were measured using survey items relating to access to areas that are interesting, attractive, with good signage relating to dog access and provisions to support dog walking [22]. Objective measures of access to public open space for dog walking was measured using a modified version of the Public Open Space Tool [25] with the addition of dog litter bags and dog-related signage [18].

**Table 1:** Univariable associations with perceived dog encouragement to walk in 629 dog owners in RESIDE Study, Perth, Western Australia (Sep 2003-Mar 2005)

Variable		Less than very often (<3pwk) *N* (%)	Very often (3 + pwk) *N* (%)	OR	95 % CI	P
Dog-related factors						
Number of dogs owned	Single	263 (56.4)	203 (43.6)	1		
	Multiple	75 (51.4)	71 (48.6)	1.23	0.85–1.78	0.28
Dog size	Small	153 (62.4)	92 (37.6)	1		
	Medium/large	181 (50.0)	181 (50.0)	1.66	1.20–2.32	**0.002**
Level of attachment to dog	Medium/low	77 (74.8)	26 (25.2)	1		
	High	261 (51.3)	248 (48.7)	2.81	1.75–4.54	**<0.001**
Spouse/partner main person in household who walks with dog	Yes	64 (66.7)	32 (33.3)	1		
	No	274 (53.1)	242 (46.9)	1.77	1.12–2.79	**0.02**
Child main person in household who walks with dog	Yes	42 (77.8)	12 (22.2)	1		
	No	296 (53.0)	262 (47.0)	3.10	1.60–6.01	**<0.001**
Owns an overweight dog	Yes	38 (58.0)	42 (42.0)	1		
	No	271 (54.4)	227 (45.6)	1.16	0.75–1.79	0.51
Affected by poor health or age of dog	Yes	148 (56.7)	113 (43.3)	1		
	No	186 (53.8)	160 (46.2)	1.13	0.82–1.56	0.47
Perceived access to Public Open Space with dog-supportive features	Poor	92 (51.7)	86 (48.3)	1		
	Good/Average	246 (56.8)	187 (43.2)	0.81	0.57–1.15	0.25
Park within 1.6 km of home with dog-supportive features	No	288 (55.9)	227 (44.1)	1		
	Yes	50 (51.5)	47 (48.5)	1.19	0.77–1.84	0.43
Cognitive factors						
Subjective norm of family, other owners and veterinarian to walking with dog daily	Neutral/Negative	276 (57.9)	201 (42.1)	1		
	Positive	59 (45.7)	70 (54.3)	1.63	1.10–2.41	**0.01**
Perceived dog-specific barriers to walking with dog daily	Ambivalent-Likely to discourage	154 (48.6)	163 (51.4)	1		
	Unlikely to discourage	180 (62.1)	110 (37.9)	1.73	1.25–2.39	**0.001**
Knowing dog enjoys going for a walk	Ambivalent-Unlikely motivation to walk with dog daily	146 (67.6)	70 (32.4)	1		
	Likely motivation to walk with dog daily	188 (48.1)	203 (51.9)	2.25	1.59–3.19	**<0.001**
I feel safe when walking with my dogs	Ambivalent-Unlikely motivation to walk with dog daily	236 (57.4)	175 (42.6)	1		
	Likely motivation to walk with dog daily	98 (50.0)	98 (50.0)	1.35	1.06–2.1.90	0.09
Perceived dog-specific behavioural outcomes from walking with dog daily	Ambivalent-Unlikely result in positive outcomes	214 (55.6)	171 (44.4)	1		
	Likely positive outcomes	117 (53.7)	101 (46.3)	1.08	0.77–1.51	0.65
Keep dog healthy	Ambivalent-Unlikely outcome of walking with dog daily	80 (77.7)	23 (22.3)	1		
	Likely outcomes of walking with dog daily	251 (50.2)	249 (49.8)	3.45	2.10–5.67	**<0.001**
Reduce the risk of dog barking	Ambivalent-Unlikely outcome of walking with dog daily	283 (56.4)	219 (43.6)	1		
	Likely outcomes of walking with dog daily	48 (47.5)	53 (52.5)	1.43	0.93–2.19	0.10
Dog might attack other dogs or people	Ambivalent-Likely outcome of walking with dog daily	233 (53.2)	196 (46.8)	1		
	Unlikely outcome of walking with dog daily	108 (58.7)	76 (42.3)	1.25	0.88–1.77	0.21
Owner demographic factors						
Gender	Male	129 (60.6)	84 (39.4)	1		
	Female	206 (52.3)	188 (47.7)	1.40	1.00–1.97	**0.05**
Age	Mean (SD) years	40.8 (10.6)	42.2 (11.4)	1.01	1.00–1.03	0.13
Children at home <18 years	Yes	206 (63.0)	121 (37.0)	1		
	No	122 (45.4)	147 (54.6)	2.05	1.48–2.85	**<0.001**
Country of origin	Australia	214 (57.5)	158 (42.5)	1		
	Other	123 (51.5)	116 (48.5)	1.27	0.92–1.77	0.14
Education	Secondary or less	138 (59.0)	96 (41.0)	1		0.24
	Trade/apprentice/certificate	128 (54.2)	108 (45.8)	1.21	0.84–1.75	0.30
	Bachelor or higher	66 (50.0)	66 (50.0)	1.44	0.94–2.21	0.10
Occupation	Management/admin	47 (51.1)	45 (48.9)	1		0.83
	Professional	89 (53.6)	77 (46.4)	0.90	0.54–1.51	0.70
	Blue collar	54 (58.7)	38 (41.3)	0.74	0.41–1.32	0.30
	Clerical/sales/service/other	78 (55.7)	62 (44.3)	0.83	0.49–1.41	0.49
	Not in workforce	65 (57.5)	48 (42.5)	0.77	0.44–1.34	0.36
Other social support for walking						
Social support from family to go walking in past month	Poor	135 (63.4)	78 (36.6)	1		
	Average/Good	201 (51.4)	190 (48.6)	1.64	1.16–2.30	**0.01**
Social support from friends to go walking in past month	Poor	287 (56.8)	218 (43.2)	1		
	Average/Good	49 (49.5)	50 (50.5)	1.34	0.87–2.07	0.18

values in bold if 0.05 or less

**Table 2:** Univariable associations with perceived dog motivation to walk in 629 dog owners in RESIDE Study, Perth, Western Australia (Sep 2003-Mar 2005)

Variable		Disagree/neutral *N* (%)	Agree *N* (%)	OR	95 % CI	P
Dog-related factors						
Number of dogs owned	Single	164 (34.2)	315 (65.8)	1		
	Multiple	49 (32.7)	101 (67.3)	1.07	0.73–1.59	0.72
Dog size	Small	101 (40.4)	149 (59.6)	1		
	Medium/large	109 (29.1)	265 (70.9)	1.65	1.18–2.31	**0.004**
Level of attachment to dog	Medium/low	61 (58.7)	43 (41.3)	1		
	High	152 (29.0)	373 (71.0)	3.48	2.26–5.37	**<0.001**
Spouse/partner main person in household who walks with dog	Yes	51 (51.0)	49 (49.0)	1		
	No	162 (30.6)	367 (69.4)	2.36	1.53–3.64	**<0.001**
Child main person in household who walks with dog	Yes	38 (69.1)	17 (31.0)	1		
	No	175 (30.5)	399 (69.5)	5.10	2.80–9.28	**<0.001**
Owns an overweight dog	Yes	42 (41.6)	49 (58.4)	1		
	No	165 (32.1)	349 (67.9)	1.51	0.97–2.33	0.07
Affected by poor health or age of dog	Yes	104 (39.0)	163 (61.0)	1		
	No	107 (30.2)	247 (69.8)	1.47	1.05–2.06	**0.02**
Perceived access to Public Open Space with dog-supportive features	Poor	52 (28.9)	128 (71.1)	1		
	Good/Average	160 (35.9)	286 (64.1)	0.73	0.50–1.06	0.10
Park within 1.6 km of home with dog-supportive features	No	180 (34.0)	349 (66.0)	1		
	Yes	33 (33.0)	67 (66.1)	1.05	0.67–1.65	0.84
Cognitive factors						
Subjective norm of family, other owners and veterinarian to walking with dog daily	Neutral/Negative	185 (37.8)	305 (62.2)	1		
	Positive	26 (19.8)	105 (80.2)	2.45	1.54–3.91	**<0.001**
Perceived dog-specific barriers to walking with dog daily	Ambivalent-Likely to discourage	87 (26.9)	236 (57.6)	1		
	Unlikely to discourage	124 (41.6)	174 (58.4)	1.93	1.38–2.71	**0.004**
Knowing dog enjoys going for a walk	Ambivalent-Unlikely motivation to walk with dog daily	116 (52.3)	106 (47.7)	1		
	Likely motivation to walk with dog daily	95 (23.8)	304 (76.2)	3.50	2.47–4.97	**<0.001**
I feel safe when walking with my dogs	Ambivalent-Unlikely motivation to walk with dog daily	161 (38.2)	260 (61.8)	1		
	Likely motivation to walk with dog daily	50 (25.0)	150 (75.0)	1.86	1.28–2.71	**<0.001**
Perceived dog-specific behavioural outcomes from walking with dog daily	Ambivalent-Unlikely result in positive outcomes	138 (34.9)	257 (65.1)	1		
	Likely positive outcomes	70 (31.5)	152 (68.5)	1.17	0.82–1.66	0.39
Keep dog healthy	Ambivalent-Unlikely outcome of walking with dog daily	63 (60.6)	41 (39.4)	1		
	Likely outcomes of walking with dog daily	145 (28.3)	368 (71.7)	3.90	2.52–6.04	**<0.001**
Reduce the risk of dog barking	Ambivalent-Unlikely outcome of walking with dog daily	186 (36.1)	329 (63.9)	1		
	Likely outcome of walking with dog daily	22 (21.6)	80 (78.4)	2.06	1.24–3.41	**0.01**
Dog might attack other dogs or people	Ambivalent-Likely outcome of walking with dog daily	139 (32.4)	290 (67.6)	1		
	Unlikely outcome of walking with dog daily	69 (36.7)	119 (63.3)	1.21	0.85–1.73	0.30
Owner demographic factors						
Gender	Male	76 (35.0)	141 (65.0)	1		0.38
	Female	136 (33.5)	270 (66.5)	1.07	0.76–1.51	0.70
Age	Mean (SD) years	41.9 (9.9)	41.3 (11.6)	1.0	0.99–1.01	0.54
Children at home <18 years	Yes	145 (43.3)	190 (56.7)	1		
	No	62 (22.5)	214 (77.5)	2.63	1.85–3.76	**<0.001**
Country of origin	Australia	136 (35.7)	245 (64.3)	1		
	Other	77 (31.2)	170 (68.8)	1.23	0.87–1.72	0.24
Education	Secondary or less	96 (39.8)	154 (60.2)	1		**0.04**
	Trade/apprentice/certificate	19 (32.4)	165 (67.6)	1.38	0.95–2.01	0.09
	Bachelor or higher	37 (27.6)	97 (72.4)	1.74	1.10–2.74	**0.02**
Occupation	Management/admin	35 (35.7)	63 (64.3)	1		
	Professional	49 (29.2)	119 (70.8)	1.35	0.79–2.29	0.27
	Blue collar	29 (30.9)	65 (69.1)	1.25	0.68–2.27	0.48
	Clerical/sales/service/other	52 (36.1)	92 (63.9)	0.98	0.58–1.68	0.95
	Not in workforce	46 (39.7)	70 (60.3)	0.86	0.49–1.47	0.55
Other social support for walking						
Social support from family to go walking in past month	Poor	97 (45.5)	116 (54.5)	1		
	Average/Good	110 (28.1)	281 (71.9)	2.14	1.51–3.03	**<0.001**
Social support from friends to go walking in past month	Poor	179 (35.4)	326 (64.6)	1		
	Average/Good	28 (28.3)	71 (71.7)	1.39	0.87–2.24	0.17

values in bold if 0.05 or less

### Statistical analysis

Chi-squared tests or t-tests and binary logistic regression analyses were used to conduct univariable analyses. Variables were then entered into the multivariable model if *P* < 0.1 in the univariable analysis. Findings are presented adjusted for the other variables.

## Results

Forty-five percent of owners reported that their dog ‘very often (3+ per week)’ ‘encouraged them to walk’ in the past month and 66 % of owners reported that they agreed with the statement ‘Having my dog(s) makes me walk more’.

### Univariable analysis

Variables associated with ‘Dog encouragement to walk’ on univariable analysis are presented in Table 1 and variables associated with ‘Dog motivation to walk’ are presented in Table 2.

### Multivariable analysis

**Fig. 2:**
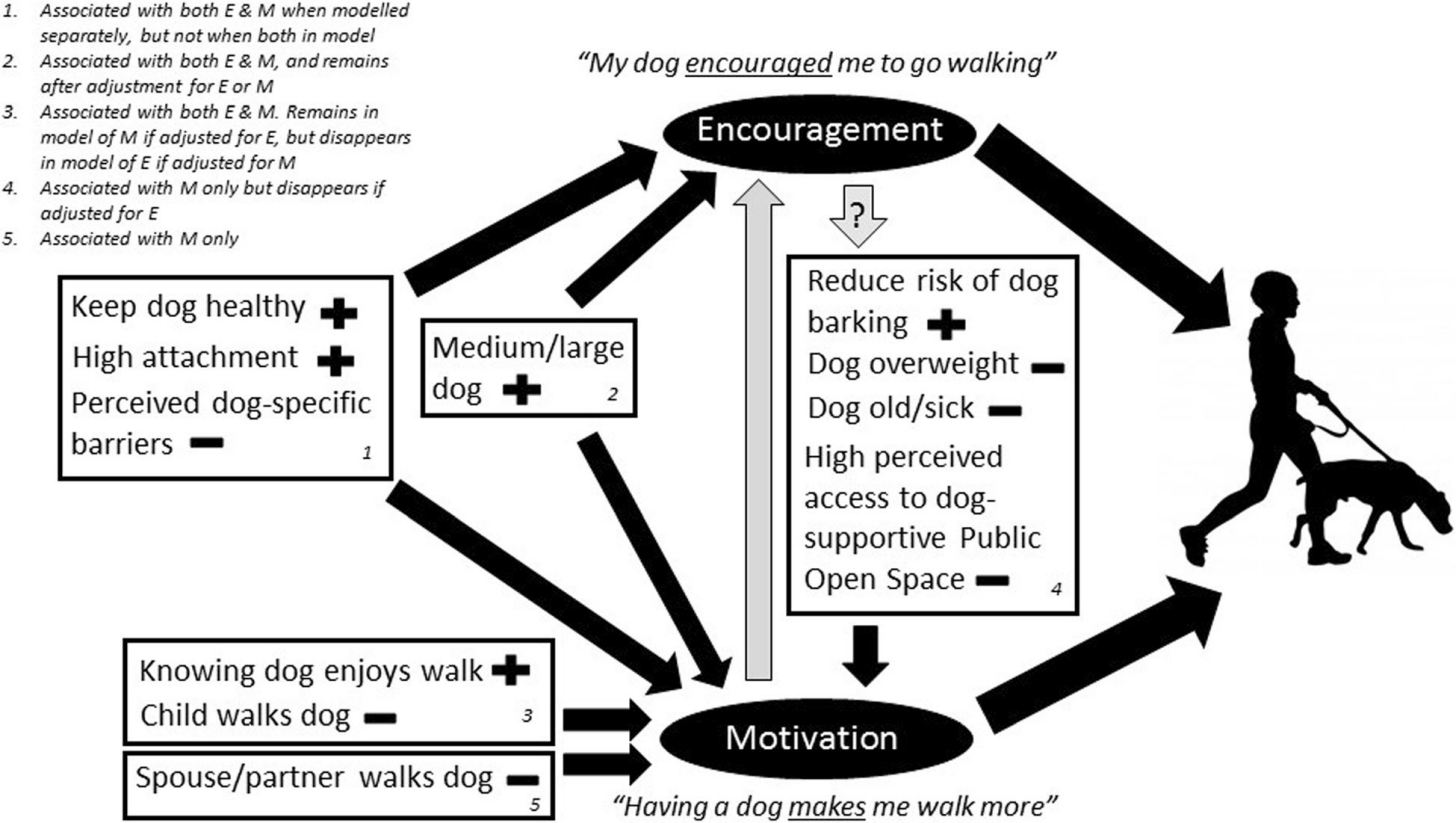
Findings from multivariable models of factors associated with encouragement and motivation provided by dogs for walking in the RESIDE dataset

Each Lassie effect outcome was modelled both with and without the presence of the other as an independent variable. When the other outcome variable was included in the multivariable model, many of the other factors became non-significant. This is to be expected as the outcomes were highly associated (OR = 10.55, 95 % CI = 6.72–16.56, *P* < 0.001). Therefore the final models are presented non-adjusted for the other outcome (Table 3). For information, size remained in both models when the other Lassie effect variable was included. A conceptual map of the findings is presented in Fig. 2.

**Table 3:** Multivariable models of perceived encouragement and motivation for dog walking in 629 dog owners in RESIDE Study, Perth, Western Australia (Sep 2003-Mar 2005)

Model outcome		Encouragement^a^			Motivation^b^		
Variable		Adj OR	95 % CI	*P*	Adj OR	95 % CI	*P*
Dog-related factors							
Dog size	Small	1			1		
	Medium/large	1.81	1.24–2.63	0.002	2.06	1.33–3.17	**0.001**
Level of attachment to dog	Medium/low	1			1		
	High	1.70	0.99–2.92	0.05	2.12	1.21–3.74	**0.01**
Spouse/partner main person in household who walks with dog	Yes	1			1		
	No	1.55	0.91–2.64	0.11	2.44	1.35–4.42	**0.003**
Child main person in household who walks with dog	Yes	1			1		
	No	2.46	1.13–5.33	0.02	5.34	2.40–11.85	**<0.001**
Owns an overweight dog	Yes				1		
	No	-	-	-	1.76	1.01–3.07	**0.05**
Affected by poor health or age of dog	Yes				1		
	No	-	-	-	1.58	1.00–2.49	**0.05**
Perceived access to POS with dog-supportive features	Poor				1		
	Good/Average	-	-	-	0.57	0.36–0.91	**0.02**
Cognitive factors							
Subjective norm of family, other owners and veterinarian to walking with dog daily	Neutral/Negative	1			1		
	Positive	1.12	0.72–1.73	0.62	1.68	0.94–2.99	0.08
Perceived dog-specific barriers to walking with dog daily	Ambivalent-Likely to discourage	1			1		
	Unlikely to discourage	1.65	1.15–2.38	0.01	1.60	1.02–2.52	**0.04**
Knowing dog enjoys going for a walk	Ambivalent-Unlikely motivation to walk with dog daily	1			1		
	Likely motivation to walk with dog daily	1.87	1.21–2.90	0.01	2.84	1.73–4.67	**<0.001**
I feel safe when walking with my dogs	Ambivalent-Unlikely motivation to walk with dog daily	1			1		
	Likely motivation to walk with dog daily	0.77	0.50–1.18	0.23	1.12	0.67–1.90	0.65
Keep dog healthy	Ambivalent-Unlikely outcome of walking with dog daily	1			1		
	Likely outcomes of walking with dog daily	2.07	1.17–3.68	0.01	1.96	1.10–3.46	**0.02**
Reduce the risk of dog barking	Ambivalent-Unlikely outcome of walking with dog daily				1		
	Likely outcomes of walking with dog daily	1.31	0.81–2.14	0.27	1.83	1.14–2.93	**0.01**
Demographic factors							
Gender	Male	1					
	Female	1.39	0.94–2.05	0.10	-	-	-
Children at home <18 years	Yes	1			1		
	No	1.68	1.16–2.45	0.01	2.12	1.35–3.28	**0.001**
Education	Secondary or less				1		0.18
	Trade/apprentice/certificate	-	-	-	1.50	0.94–2.41	0.09
	Bachelor or higher	-	-	-	1.53	0.85–2.75	0.16
Other social support for walking							
Social support from family to go walking in past month	Poor	1			1		
	Average/Good	1.76	1.19–2.58	0.004	2.58	1.66–4.01	**<0.001**

^a^ *N* = 573, Hosmer-Lemeshow 0.82, Classification table percentage correct = 66.1

^b^ *N* = 559, Hosmer-Lemeshow 0.33, Classification table percentage correct = 76.7

values in bold if 0.05 or less

#### Dog encouragement to walk

There was evidence for an independent association between the outcome ‘Dog encouragement to walk’ and dog size (medium/large vs small OR = 1.81, 95 % CI = 1.24–2.63, *P* = 0.002) and level of attachment to dog (high vs medium/low OR = 1.70, 95 % CI = 0.99–2.92, *P* = 0.05) (Table 3). Associated dog-related cognitive factors included: knowing dog enjoys going for a walk (‘likely motivation to walk with dog daily’ vs ‘ambivalent-unlikely motivation to walk with dog daily’ OR = 1.87, 95 % CI = 1.21–2.90, *P* = 0.01); keep dog healthy (‘likely motivation to walk with dog daily’ vs ‘ambivalent-unlikely motivation to walk with dog daily’ OR = 2.07, 95 % CI = 1.17–3.68, *P* = 0.01); and perceived dog-specific barriers to walking with dog daily (‘unlikely to discourage’ vs ‘ambivalent-likely to discourage’ OR = 1.65, 95 % CI = 1.15–2.38, *P* = 0.01) (Table 3). There was also an association with: presence of children at home <18 years (no vs yes OR = 1.68, 95 % CI = 1.16–2.45; *P* = 0.01); child main person in household who walks with dog (no vs yes OR = 2.46, 95 % CI = 1.13–5.33, *P* = 0.02); and social support from family to go walking in past month (average/good vs poor OR = 1.76, 95 % CI = 1.19–2.58, *P* = 0.004) (Table 3).

The model for encouragement found that inclusion of motivation accounted for all other variables except size.

#### Dog motivation to walk

There was evidence for an independent association between the outcome ‘Dog motivation to walk’ and: dog size (medium/large vs small OR = 2.06, 95 % CI = 1.33–3.17, *P* = 0.001); level of attachment to dog (high vs medium/low OR = 2.12, 95 % CI = 1.21–3.74,*P* = 0.01); owns an overweight dog (no vs yes OR = 1.76, 95 % CI = 1.01–3.07, *P* = 0.05); and affected by poor health or age of dog (no vs yes OR = 1.58, 95 % CI = 1.00–2.49, *P* = 0.05). Other dog-related cognitive factors associated with dog motivation to walk were: knowing dog enjoys going for a walk (‘likely motivation to walk with dog daily’ vs ‘ambivalent-unlikely motivation to walk with dog daily’ OR = 2.84, 95 % CI = 1.73–4.67, *P* < 0.001); keep dog healthy (‘likely motivation to walk with dog daily’ vs ‘ambivalent-unlikely motivation to walk with dog daily’ OR = 1.96, 95 % CI = 1.10–3.46, *P* = 0.02); reduce the risk of dog barking (‘likely outcome of walking with dog daily’ vs ‘ambivalent-unlikely outcome of walking with dog daily’ OR = 1.83, 95 % CI = 1.14–2.93, *P* = 0.01).; and perceived dog-specific barriers to walking with dog daily (‘unlikely to discourage’ vs ‘ambivalent-likely to discourage’ OR = 1.60, 95 % CI = 1.02–2.52, *P* = 0.04)). Perceived access to Public Open Space with dog-supportive features was associated with lower motivation (good/average vs poor OR = 0.57, 95 % CI = 0.36–0.91, *P* = 0.02). Further, there was an association with: presence of children at home <18 years (no vs yes OR = 2.12, 95 % CI = 1.35–3.28, *P* = 0.001); spouse/partner main person in household who walks with dog (no vs yes OR = 2.44, 95 % CI = 1.35–4.42, *P* = 0.003), or child main person in household who walks with dog (no vs yes OR = 5.34, 95 % CI = 2.40–11.85, *P* < 0.001; and social support from family to go walking in past month (average/good vs poor OR = 2.58, 95 % CI = 1.66–4.01, *P* < 0.001) (Table 3).

The model for motivation found that encouragement inclusion rendered the other variables insignificant except size, knowing dog enjoys going for a walk, spouse/partner main person in household who walks with dog, child main person in household who walks with dog, children at home <18 years, and social support from family to go walking in past month.

## Discussion

Our findings elucidate how the motivation, encouragement, obligation or social support for walking that an individual pet dog can provide is important to understanding dog walking behaviour. Both Lassie effect outcomes ‘Dog encouragement to walk’ and ‘Dog motivation to walk’ appear to be associated with the key variables of: dog size; level of attachment to dog; perceived dog-specific barriers to walking with dog daily; knowing dog enjoys going for a walk; belief walking keeps dog healthy; children at home <18 years; the child being the main person who walks with the dog; and social support from family to go walking in past month. In addition, ‘Dog motivation to walk’ only was positively associated with the belief that walking reduces barking, and negatively with; dog overweight; dog too old/sick; spouse/partner main person who walks with the dog; and increased perceived access to public open spaces with dog-supportive features. Thus this supports our hypothesis that dog-related factors influence the dog-human relationship through the encouragement and motivation certain dogs provide for walking, which in turn (based on existing studies) influences dog walking behaviour.

Our findings suggest that larger dogs appear to encourage and motivate dog walking compared with smaller dogs. Likewise having a strong attachment to the dog is associated with feelings of encouragement and motivation to walk the dog. A few other studies have also found that dog size (larger vs smaller) [26, 27] and stronger attachment [10] are associated with higher levels of dog walking behaviour. However, the majority of other studies exploring dog-related factors found no association with walking [9]. Here we demonstrate this is because the mechanism through which dog size and attachment influence dog walking behaviour is through the Lassie effect.

The belief that walking with a dog daily keeps the dog healthy was associated with both increased motivation and perceived encouragement provided by the dog for walking. This supports other evidence showing that owner’s perceptions about the benefits of dog walking for their dog has a role to play in the pathway to changing dog walking behaviour [16, 20].

We found that perceived dog-specific barriers to walking with a dog daily was negatively associated with both Lassie effect outcomes. Perceived dog specific barriers to daily dog walking includes difficulties in walking two dogs (as opposed to one), owners’ fear of other dogs and concerns a person’s own dog will be unfriendly and difficult to control while walking. These perceived barriers reduce feelings of encouragement and motivation to walk the dog. This agrees with other studies showing that dog behaviour problems can negatively influence walking [9], however our findings highlight that the pathway is through reduced encouragement and motivation to walk the dog. Interestingly, some aspects of dog behaviour such as fear that a person’s own dog may attack other dogs, or perceiving positive behavioural outcomes from walking the dog, were not associated with either ‘Lassie’ effect outcome. This area requires deeper investigation.

Interestingly, people who experienced the Lassie effect from their dogs also perceived that their family provided social support for walking, suggesting that they may enjoy walking together as a family unit. There has been little research into the influence of social support from family members or friends on dog walking behaviour [9]. One Australian study reported that 68 % of dog-owning children’s families reported walking the dog as a family at least once a month [28]. Perceived social support for walking provided by other family members appears to positively influence an owner’s perceived social support and motivation/obligation to walk their dog and suggests that the support that a dog and a family member provide for walking may be entwined. However, we also observed that if someone else in the household reported being the main person walking the dog (e.g., a partner/spouse or child) this was associated with lower odds of reporting the Lassie effect outcomes. This is logical as encouragement and motivation for dog walking is unlikely to be felt if somebody else in the household usually does the dog walking without you. This highlights that future studies should determine not just dog ownership but who walks the dog and how this is shared with other family members.

Finally, we found that having children at home may be a barrier to experiencing the Lassie effect. This may be due to the heightened perceived lack of time for physical activity of parents of children [29, 30]. Previous studies have shown mixed findings of the effect of increased household size/or having dependents on dog walking behaviour [9]. Further research is needed to understand the relationships between the factors associated with the Lassie effect and dog walking behaviour in families with children.

While many factors were associated with both dog encouragement and motivation to walk, there were some differences between these measures of the Lassie effect. The belief that walking with a dog daily reduces the risk of barking was associated with increased motivation/obligation but not perceived encouragement for dog walking. This supports other evidence showing that owners perceive a number of dog behaviour benefits from walking their dog [16] and this can be an incentive to walking with your dog. However it is unknown why it might affect perceived motivation but not encouragement provided by the dog to walk. A plausible explanation may be that reduced barking is a benefit of dog walking that is only realised once walking is established. Thus it may be a construct of the motivation to maintain dog walking over time rather than initially feeling encouraged to take the dog out for a walk. Similarly, the influence of old/sick dogs was associated with reduced motivation but not perceived encouragement to walk the dog. This suggests that having a sick/old dog is a barrier to feeling motivated or obliged to walk the dog, regardless of how much encouragement or perceived social support for walking the presence of a dog may provide. Owners of overweight dogs also showed reduced motivation to walk their dog.

Overall, these differences highlight that the two outcomes of The Lassie effect modelled may refer to different aspects of dog walking, or alternatively, the difference in findings may be reflecting differences in the wording of the two question answers and they are essentially measuring the same construct. However, two previous studies have shown that the two constructs were found to be independently associated with dog walking outcomes [18, 19]. In the current study where we modelled motivation and encouragement as outcomes (rather than dog walking as per previous studies), the addition of motivation to the encouragement model rendered many of the other variables obsolete. However when the encouragement construct was added to the motivation model, a number of variables remained. This suggests that the encouragement and motivation variables do not simply replace each other in the models. This could plausibly suggest that the wording of the ‘motivation’ construct partly includes the idea of encouragement but also something more, we suggest being that extra sense of ‘obligation’ or ‘responsibility’ adding to encouragement to provide specific motivation for walking with a dog.

Looking closer at differences in the data provides further support for this. Knowing that your dog also enjoys the walking experience was associated with both outcomes, however comparison of the models with and without the other Lassie effect variable suggests that knowing the dog enjoys going for a walk may be more important to motivation to keep walking than encouragement to go walking. This may be because you have to already be walking the dog before you can perceive that it enjoys it. Comparison of the models with and without the other Lassie effect variables also suggested that the role of spouses or children walking the dog may be more directed to motivation than encouragement. This could be explained by the idea that a dog may behave in a way that encourages walking but in reality someone else will do it. Further studies are required to examine whether the differences in our two Lassie effect outcomes may relate to initiation (encouragement) and maintenance (motivation) of dog walking. Importantly, further research is required to understand and assist with consistent terminology associated with the effect of dog-related variables on the initiation (encouragement) and maintenance (motivation) of dog walking.

In addition, perceived access to public open space with dog supportive features contributed to perceived motivation but not encouragement to dog walk. Even more interestingly, the direction of the effect was opposite to what might be expected, with a higher perceived access to places with dog supportive features (e.g., ‘poo’ bins and dog-related signage) associated with lower odds of reporting high motivation to dog walk. We hypothesise that perceived access to areas with dog-supportive features may be less relevant if the motivation to walk the dog is already high, as the dog will be walked regardless of whether there are places with dog-supportive features to walk. In fact those who feel having a dog makes them walk more may perceive that their local walking areas are actually not very dog supportive because they observe them directly when they are out walking.

Our findings have a number of implications for the future design of interventions to increase dog walking and community levels of physical activity. Findings confirm that dog-specific and dog-human relationship factors are important to perceptions regarding the need to walk the dog. Interventions should aim to target the perceived encouragement and motivation the pet dog can provide for walking. This may be through changing perceptions of the amount of exercise smaller and older dogs require, and how much dogs enjoy walking. Interventions may also be able to target the attachment to the dog, perhaps by increasing this through participation in training exercises or activities with the dog. Furthermore, tackling dog-specific behavioural barriers through dog and owner education and training may also naturally have this effect of strengthening the owner-dog relationship as well as addressing specific barriers to walking. Interventions could also perhaps specifically be targeted at owners of small dogs and dogs with walking-related behavioural problems. They could also be directed at families with children who are likely to feel less encouraged/obligated to walk the dog because of time pressures, as these may be hard to reach groups but with the most to gain. It is clear that for the success of such interventions, interdisciplinary collaboration between health promotion practitioners, veterinarians and dog behaviour experts is key.

This study appears to be the first to unpack how dog and owner-related factors influence the single largest factor correlated with dog walking behaviour – the perceived encouragement/social support/motivation/obligation provided by the dog to walk. Another strength of this study is that the sample is population rather than convenience-based. However it is limited by its specificity to people who had recently moved into new housing areas, and thus may not be representative of all dog owners. Factors not measured in this study such as dog breed and training, as well as socialization, could be relevant and should be considered in future research. Why factors such as dog size and age influence beliefs about exercise requirements requires further exploration. The hypothetical pathway to dog walking behaviour described requires confirmation using mediation analyses. Finally, future studies may wish to examine if and how the perceived social support/motivation/obligation provided by a dog to walk changes according to the initiation and maintenance of dog walking behaviour.

## Conclusions

Our findings suggest a pathway where dog and owner factors affect the dog-owner relationship in terms of encouragement and motivation to walk the dog, which then affects dog walking behaviour (as shown in previous studies). Interventions should target owners of small dogs and dogs with behavioural problems when walking, and families with children who are likely to feel less encouraged/motivated to walk the dog. Future studies need to investigate in more detail how the dog-owner relationship of different dog and owner demographics leads to walking behaviour. They should also investigate whether initiation and maintenance of dog walking is affected by different factors. Further investigation is also required into the life stage of families and how this affects the dog-owner relationship and dog walking behaviour. Future studies should be prospective and use objective measures of walking where possible.
